# Neural diffusivity and pre-emptive epileptic seizure intervention

**DOI:** 10.1371/journal.pcbi.1008448

**Published:** 2020-12-01

**Authors:** Erik D. Fagerholm, Chayanin Tangwiriyasakul, Karl J. Friston, Inês R. Violante, Steven Williams, David W. Carmichael, Suejen Perani, Federico E. Turkheimer, Rosalyn J. Moran, Robert Leech, Mark P. Richardson

**Affiliations:** 1 Department of Neuroimaging, King’s College London, London, United Kingdom; 2 Department of Basic and Clinical Neuroscience, King's College London, London, United Kingdom; 3 School of Biomedical Engineering & Imaging Sciences, King’s College London, London, United Kingdom; 4 Wellcome Centre for Human Neuroimaging, University College London, London, United Kingdom; 5 School of Psychology, University of Surrey, Guildford, United Kingdom; 6 Developmental Neurosciences, University College London, London, United Kingdom; 7 UCL Great Ormond Street Institute of Child Health, University College London, London, United Kingdom; 8 Centre for Epilepsy, King's College Hospital, London, United Kingdom; Ghent University, BELGIUM

## Abstract

The propagation of epileptic seizure activity in the brain is a widespread pathophysiology that, in principle, should yield to intervention techniques guided by mathematical models of neuronal ensemble dynamics. During a seizure, neural activity will deviate from its current dynamical regime to one in which there are significant signal fluctuations. *In silico* treatments of neural activity are an important tool for the understanding of how the healthy brain can maintain stability, as well as of how pathology can lead to seizures. The hope is that, contained within the mathematical foundations of such treatments, there lie potential strategies for mitigating instabilities, e.g. via external stimulation. Here, we demonstrate that the dynamic causal modelling neuronal state equation generalises to a Fokker-Planck formalism if one extends the framework to model the ways in which activity propagates along the structural connections of neural systems. Using the Jacobian of this generalised state equation, we show that an initially unstable system can be rendered stable via a reduction in diffusivity–i.e., by lowering the rate at which neuronal fluctuations disperse to neighbouring regions. We show, for neural systems prone to epileptic seizures, that such a reduction in diffusivity can be achieved via external stimulation. Specifically, we show that this stimulation should be applied in such a way as to temporarily mirror the activity profile of a pathological region in its functionally connected areas. This counter-intuitive method is intended to be used pre-emptively–i.e., in order to mitigate the effects of the seizure, or ideally even prevent it from occurring in the first place. We offer proof of principle using simulations based on functional neuroimaging data collected from patients with idiopathic generalised epilepsy, in which we successfully suppress pathological activity in a distinct sub-network prior to seizure onset. Our hope is that this technique can form the basis for future real-time monitoring and intervention devices that are capable of treating epilepsy in a non-invasive manner.

## Author summary

Epilepsy is a disease that affects over 50 million people worldwide. Current treatments include dangerous surgical procedures in which brain connections are severed, or even in which entire problem brain regions are removed. Pharmaceutical options are available, but only about one third of patients are responsive. However, even in these cases the drugs can cause such severe side effects that the patients sometimes choose to suffer seizures. We are proposing an innovative treatment of epilepsy that could be achieved by using non-invasive electrical stimulation. Specifically, we show that stimulation should be applied in such a way as to mirror the activity in a problem brain region, by targeting its neighbouring areas. This counterintuitive approach is based on a mathematical model in which this mirroring strategy is applied pre-emptively, i.e. long before the seizure has a chance to set in. The hope is that future clinical trials will be able to use this model to lessen the effect of seizures, or even prevent them from occurring in the first place.

## Introduction

There is ongoing interest in using stimulation techniques [1–3] to treat epilepsy by directly modifying the physiological states of neural systems [4,5]. However, the three basic questions of *when*, *where* and *how* to stimulate for maximum clinical efficacy remain unanswered [6–8]. As such, there is a pressing need for computational frameworks that are able to model the effects of external stimulation and to guide the development of optimal intervention protocols. Several mathematical models have been put forward to describe the phenomenology of seizure initiation [9] and to predict neurosurgical outcome [10,11], primarily using electroencephalography (EEG) and electrocorticography (ECoG) in patients with focal epilepsy. In this study, we propose a novel approach for pre-emptive seizure intervention, based on the computational framework of dynamic causal modelling (DCM) [12] and tested with a combination of EEG and functional magnetic resonance imaging (fMRI) data in patients with idiopathic generalised epilepsy (IGE).

DCM provides a powerful analytical tool for inferring latent structure from blood-oxygen-level-dependent (BOLD) time series by optimising the parameters, such as intrinsic connectivity and external driving inputs, of a generative model. DCM rests upon the neuronal state equation which, in its simplest mathematical form, is written as a linear ordinary differential equation describing the ways in which neuronal signals change with respect to time. Here, we show that the neuronal state equation can be generalised to account for the ways in which signals change–not only with respect to time–but also with respect to the structure of the network within which the signals are constrained to propagate.

This paper comprises three sections.

In the first section, we show that the additional structural component of the generalised neuronal state equation conforms to a diffusion process, which facilitates the suppression of activity via gradient modulation in brain regions prone to epileptic seizures. Furthermore, the structural term renders the mathematical form of the generalised neuronal state equation identical to that of the discretized Fokker-Planck equation. The latter is a partial differential equation that is used to describe the probabilistic evolution of a system, initially studied in the context of Brownian motion [13], and increasingly used in the modelling of neural systems [14,15].

In the second section, we provide construct validation that the ensuing model provides a better description (in terms of its variational free energy) of resting state fMRI. Specifically, we show that time series in both epilepsy patients and healthy control subjects are better modelled by the Fokker-Planck equation, compared with the classic (non-structural) neuronal state equation.

In the third section, we show that an initially unstable region can be pushed into a stable regime by virtue of a reduction in network diffusivity. We demonstrate that such a reduction can be achieved by using external stimulation to mirror seizure activity in the area(s) that are functionally connected to a pathological brain region. We report a series of simulations incorporating individualised EEG and fMRI data collected in patients with idiopathic generalised epilepsy (IGE) and show promising evidence that electrical stimulation is a viable method for suppressing epileptic seizures.

## Methods

### Ethics statement

The study was approved by the Riverside Research Ethics Committee (12/LO/2005) and all participants signed a written informed consent form prior to the study, according to the declaration of Helsinki (2013).

### The generalised neuronal state equation

Causal models can be considered as the evolution of states *x* as a static function of themselves. For instance, for the *i*^*th*^ region:

$$x_{i}=f(x,v,\theta ),$$ (1)

where *f* is a nonlinear function, *v* are external inputs, and *θ* are model parameters–usually interpreted in terms of connectivity or rate constants. DCM extends the assumptions in Eq (1) by considering the ways in which states change with respect to time, so that:

$$\frac{dx_{i}}{dt}=f(x,v,\theta )$$ (2)

which reduces to Eq (1) in the limit that inputs *v* vary slowly relative to the states *x*.

Extrapolating the logical progression from Eq (1) to Eq (2), we can define a generalised neuronal state equation in which states may vary, not just with respect to time as in Eq (2), but with respect to any arbitrary number of dimensions *μ*, such that:

$$\sum_{j=1}\frac{dx_{i}}{d\mu_{j}}=f(x,v,\theta )$$ (3)

**Fig 1:**
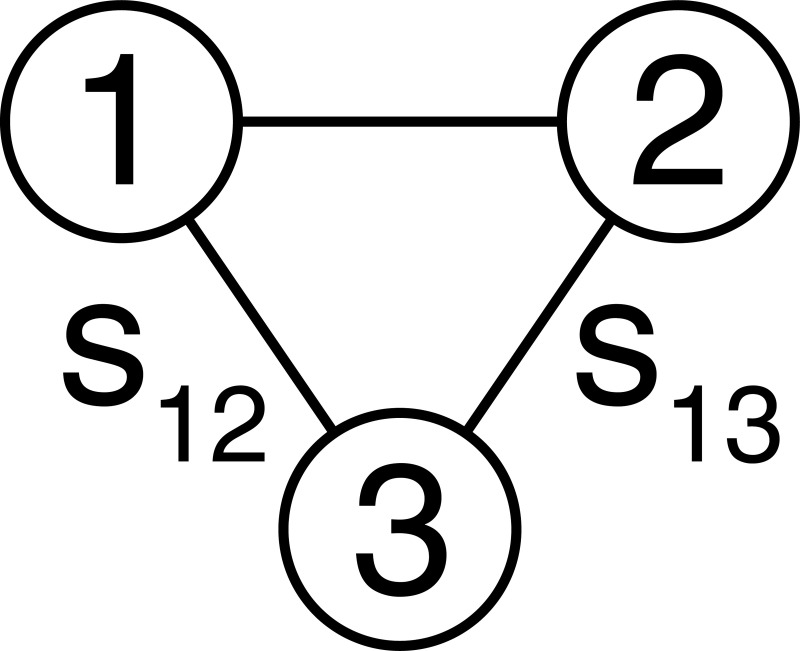
Three-node network. The structural connection between nodes 1 and 2 is given by s_12_ and the structural connection between nodes 1 and 3 is given by s_13_.

For example, if we consider the simple three-node network in Fig 1.

We describe the evolution of the first node using Eq (3) as follows:

$$\sum_{j=1}^{3}\frac{dx_{1}}{d\mu_{j}}=f(x,v,\theta )$$ (4)

We then retain time as the first dimension (*μ*_1_ = *t*) on the left-hand side and assign the remaining two terms to the rate of change of states along the structural dimensions of the network, such that:

$$\frac{dx_{1}}{dt}+\sigma \left(\frac{dx_{1}}{ds_{12}}+\frac{dx_{1}}{ds_{13}}\right)=f(x,v,\theta ),$$ (5)

where *σ* is a (rate) constant of proportionality with dimensions *T*^−1^; and e.g. $\frac{dx_{1}}{ds_{12}}$ describes the rate of change of the first node’s activity along the edge connecting the first and second nodes. Intuitively, this kind of extension can be thought of as generalising the dynamics of any one point in the brain to a partial differential equation that models the spatiotemporal dynamics (e.g., neuronal field models). However, here, instead of three spatial dimensions, we assign one dimension to each inbound edge for every node within the graph.

We write the general form of Eq (5) as follows for the *i*^*th*^ node in a network of *N* regions:

$$\frac{dx_{i}}{dt}+\sigma \sum_{j=1}^{N}k_{ij}\frac{dx_{i}}{ds_{ij}}=f(x,v,\theta )$$ (6)

where *k*_*ij*_ is the adjacency matrix element connecting the *i*^*th*^ and *j*^*th*^ regions, the inclusion of which ensures that only nearest neighbours in the network are taken into account. Note that in the context of neural systems, the definition of nearest neighbours may also be taken to mean regions that are structurally connected via white matter tracts. This means that any given region or node can have more neighbours than if we were modelling the cortical sheet as a two-dimensional Markov field.

The structural gradients $\frac{dx_{i}}{ds_{ij}}$ in Eq (6) can be discretized by expressing them in terms of the difference in state values (at a given time) at the *i*^*th*^ and *j*^*th*^ nodes, such that:

$$\frac{dx_{i}}{ds_{ij}}\to x_{j}-x_{i},$$ (7)

which, together with Eq (6), tells us that:

$$\frac{dx_{i}}{dt}=f(x,v,\theta )+\sigma \sum_{j=1}^{N}k_{ij}(x_{i}-x_{j})$$ (8)

where we see that, in the limit of zero structural gradients (*x*_*i*_ = *x*_*j*_), the second term on the right-hand side vanishes and Eq (8) reduces to the original DCM neuronal state equation in Eq (2). In other words, every node behaves in the same way and can be described with an ordinary differential equation. In summary, we obtain a third level of neuronal state equations building on the simple causal models in Eq (1), through the classical DCM in Eq (2), and ending with the generalised form in Eq (8), with each level reducing to the former in the appropriate limiting cases.

### DCM and the Fokker-Planck equation

The second term on the right-hand side of Eq (8) can be re-written as follows:

$$\sum_{j=1}^{N}k_{ij}(x_{i}-x_{j})=x_{i}\sum_{j=1}^{N}k_{ij}-\sum_{j=1}^{N}k_{ij}x_{j}=x_{i}d_{i}-\sum_{j=1}^{N}k_{ij}x_{j}=\sum_{j=1}^{N}(\delta_{ij}d_{i}-k_{ij})x_{j}=\sum_{j=1}^{N}l_{ij}x_{j}$$ (9)

where *d*_*i*_ is the degree of the *i*^*th*^ node; *δ*_*ij*_ is the Kronecker delta function, which equals unity when *i* = *j*, and otherwise equals zero; and *l*_*ij*_ is the graph Laplacian matrix element connecting the *i*^*th*^ and *j*^*th*^ nodes, where the graph Laplacian matrix *L* is defined as the difference between the degree matrix *D* and the adjacency matrix *K*, such that *L* = *D*−*K*.

Using Eq (9), we can write Eq (8) as follows:

$$\frac{dx_{i}}{dt}=f(x,v,\theta )+\sigma \sum_{j=1}^{N}l_{ij}x_{j}$$ (10)

and as the graph Laplacian is the discretized version of the Laplace operator [16], the second term on the right-hand side describes a diffusion process, hence lending an interpretation to *σ* as a diffusion coefficient. Therefore, Eq (10) takes the form of the discretized Fokker-Planck equation, with a drift term: *f*(*x*,*v*,*θ*) and a diffusion term: $\sigma \sum_{j=1}^{N}l_{ij}x_{j}$.

### Linear stability analysis

In order to model neural time series, we assume first order interactions, which means Eq (8) can be written as:

$$\frac{dx_{i}}{dt}=\sum_{j=1}^{N}p_{ij}x_{j}+\sigma \sum_{j=1}^{N}k_{ij}(x_{i}-x_{j})+\sum_{j=1}^{M}q_{ji}v_{j}+\omega^{\left(i\right)}$$ (11)

where the matrix element *p*_*ij*_ reduces to the DCM intrinsic coupling matrix element *a*_*ij*_ in the limiting case of zero structural gradients (*x*_*i*_ = *x*_*j*_); the matrix element *q*_*ji*_ reduces to the DCM extrinsic coupling matrix element *c*_*ji*_ in the limiting case of zero structural gradients (*x*_*i*_ = *x*_*j*_); and *ω*^(*i*)^ are non-Markovian fluctuations in the *i*^*th*^ region’s activity [17]. These fluctuations model deviations from the linear flow of states under an adiabatic approximation. In other words, we assume a centre manifold for the dynamics, which are linear and assign fluctuations tangential to the manifold to *ω*^(*i*)^, which decay rapidly and return to the manifold under the centre manifold theorem [18].

The stability of a linear time invariant (LTI) system [19] is not dependent upon external driving inputs, which means that these do not affect system stability or resilience to perturbations. Therefore, if we wish to determine the long-term stability of a dynamical system, we need only to consider its Jacobian. Therefore, we can re-write Eq (11) by retaining only the first two (stability-relevant) terms, such that:

$$\frac{dx_{i}}{dt}=\sum_{j=1}^{N}p_{ij}x_{j}+\sigma \sum_{j=1}^{N}k_{ij}(x_{i}-x_{j})=\sum_{j=1}^{N}\left((p_{ij}-\sigma k_{ij})xj+\sigma k_{ij}x_{i}\right),$$ (12)

which we can write out explicitly for the three-node network in Fig 1 as follows:

$$\left[\begin{array}{c} {\dot{x}}_{1}\\ {\dot{x}}_{2}\\ {\dot{x}}_{3} \end{array}\right]=\left[\begin{array}{ccc} p_{11}+\sigma (k_{12}+k_{13}) & p_{12}-\sigma k_{12} & p_{13}-\sigma k_{13}\\ p_{21}-\sigma k_{21} & p_{22}+\sigma (k_{21}+k_{23}) & p_{23}-\sigma k_{23}\\ p_{31}-\sigma k_{31} & p_{32}-\sigma k_{32} & p_{33}+\sigma (k_{31}+k_{32}) \end{array}\right]\left[\begin{array}{c} x_{1}\\ x_{2}\\ x_{3} \end{array}\right].$$ (13)

The Jacobian of this generalised DCM can thus be written as follows for a network comprising *N* regions:

$$J=[\begin{array}{ccc} p_{11}+\sigma \sum_{j\neq 1}^{N}k_{1j} & \cdots & p_{1N}-\sigma k_{1N}\\ \vdots & \ddots & \vdots \\ p_{N1}-\sigma k_{N1} & \cdots & p_{NN}+\sigma \sum_{j\neq N}^{N}k_{Nj} \end{array}].$$ (14)

### Diffusion and stability

In 1953, Turing showed that an initially stable dynamical system can be rendered unstable by virtue of a diffusion mechanism, allowing for the emergence of spatial inhomogeneities–now known as Turing patterns [20]. Here, we proceed via similar logic, except we begin with the opposite premise and ask the following question: can we push an initially unstable system (such as a neural system prone to seizures) into a stable regime by altering the system’s diffusivity?

To answer this, we multiply the diffusion coefficient *σ* by a constant *α*:

σ→ασ. (15)

By multiplying *σ* by some unknown quantity *α* in this way we can determine–in the subsequent linear stability analysis–whether this change should act in a way as to increase or decrease diffusivity in order to render an initially unstable system stable.

If the system in Eq (14) is initially unstable then at least one eigenvalue has a positive Real component. This is guaranteed to be the case when the trace (the sum of the eigenvalues) is positive:

$$trJ=p_{11}+\sigma \sum_{j\neq 1}^{N}k_{1j}+\cdots +p_{NN}+\sigma \sum_{j\neq N}^{N}k_{Nj}>0,$$ (16)

We then transform Eq (14) via Eq (15) to obtain:

$$J\prime =[\begin{array}{ccc} p_{11}+\alpha \sigma \sum_{j\neq 1}^{N}k_{1j} & \cdots & p_{1N}-\alpha \sigma k_{1N}\\ \vdots & \ddots & \vdots \\ p_{N1}-\alpha \sigma k_{1N} & \cdots & p_{NN}+\alpha \sigma \sum_{j\neq 1}^{N}k_{Nj} \end{array}],$$ (17)

which has been rendered stable due to the altered diffusion coefficient.

Stability implies that all the eigenvalues now have negative Real components, which in turn means that the trace must now be negative:

$$trJ^{\prime }=p_{11}+\alpha \sigma \sum_{j\neq 1}^{N}K_{1j}+\cdots +p_{NN}+\alpha \sigma \sum_{j\neq 1}^{N}K_{Nj}<0,$$ (18)

which, together with Eq (16), means that:

$$trJ^{\prime }=trJ+(a-1)\sigma \left(\sum_{j\neq 1}^{N}K_{1j}+\cdots +\sum_{j\neq 1}^{N}K_{Nj}\right)<0.$$ (19)

We then know from Eq [16] that *trJ*>0. Furthermore, we know that diffusion coefficients are necessarily positive, given that they play the role of rate constants [21] and also that the adjacency matrix elements *k* are either 0 or 1. Therefore, the only way in which Eq (19) can be satisfied is if:

α−1<0⟹α<1, (20)

i.e. an initially unstable system can be rendered stable by virtue of a reduction in the diffusion coefficient.

### Diffusivity can be altered via external driving inputs

In practice it is not possible to change the diffusion coefficient as in Eq (15), due to the fact that it is an intrinsic property of the dynamical system described by Eq (14). However, as the diffusion coefficient quantifies diffusivity via Fick’s law [22], we recover an important piece of information from Eq (20); namely, that if we want to push a system toward stability, we must act so as to decrease diffusivity.

If we look at the governing equation of motion [11], we note that the diffusion term $\sigma \sum_{j=1}^{N}k_{ij}(x_{i}-x_{j})$ comprises three factors: 1) the diffusion coefficient *σ*, which we noted above cannot be changed; 2) the adjacency matrix element *k*_*ij*_ which, similar to *σ*, is intrinsic to the system and is therefore also unchangeable, without resorting to severing connections e.g. via surgical intervention; and finally 3) the gradient (*x*_*i*_−*x*_*j*_), which is the only factor that can be decreased by applying external driving inputs in a way as to force *x*_*j*_ to mirror the activity profile of *x*_*i*_ as closely as possible–hence decreasing diffusivity.

Note that this discovery, i.e. that the gradients are the only alterable aspect of the equation of motion, follows directly from the generalisation of the DCM neural state equation. It should also be noted that manipulating the gradients in this way has no effect on the eigenvalues of the Jacobian and thus no effect on the intrinsic stability of the system. Instead, what we are proposing is that it should in practice be possible to induce a form of temporary ‘pseudo stability’ in strategic locations via externally forced gradient minimization.

**Fig 2:**
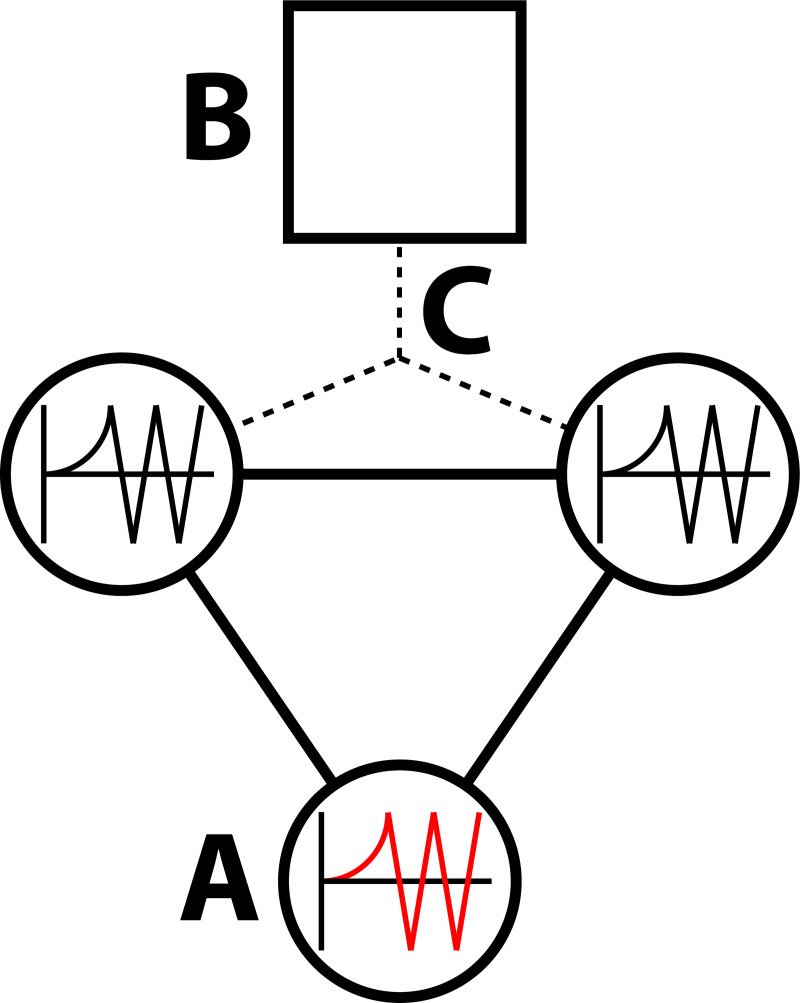
Gradient reduction via stimulation. **A)** The seizure-prone node. This displays the activity shown in the red graph therein. **B)** External driving input is applied in such a way as to allow the two neighbouring nodes to mirror the activity of the seizure-prone node in A). **C)** Extrinsic coupling to the two regions neighbouring A).

Let us now consider the same three-node system shown in Fig 1 and assume that the bottom node is prone to instabilities (Fig 2A). In the case of epilepsy, we therefore propose a form of intervention in which we apply external driving input (Fig 2B) in such a way as to allow the two neighbouring nodes (Fig 2C) to mirror the activity in the region that is prone to seizures.

In summary, we have derived a generalised DCM of neuronal activity that can generate empirical timeseries. We now proceed to estimate the parameters of the DCM using empirical timeseries from epilepsy patients. Equipped with these parameters, we can then evaluate the Jacobian and simulate the effects of an intervention that moves the ensemble or population dynamics implicit in Eq (11) from a regime of instability (i.e., seizure activity) to one of stability.

### The seizure network

In all analyses presented here, we use a network comprising the following regions: frontal mid, frontal mid orbital, precuneus, and thalamus. This network is known to play an important role in generalised spike and wave (GSW) discharges [23] and, for simplicity, we will refer to it henceforth as the ‘seizure network’.

### Participants and data acquisition

We analyse data recorded from 15 patients (6 male) with juvenile myoclonic epilepsy (JME) with a mean age of 24.5 years and no structural differences found between patients. In addition, we analyse data from 15 age-matched healthy controls (5 male) with a mean age of 25.2 years (see S1 Table). The data were acquired at the Institute of Psychiatry Psychology and Neuroscience (IoPPN), King’s College London. The patients did not have any neurological diagnoses other than epilepsy and had no history of drug or alcohol misuse. All participants underwent a resting state simultaneous EEG-fMRI and a diffusion tensor imaging (DTI) session, with 32 directions and b = 1500 s/mm^2^. We do not obtain directional information regarding structural connectivity via DTI and subsequent adjacency matrices are therefore symmetric. A 3T scanner (MR750, GE Healthcare) was used to acquire 300 echo-planer images (3.3×3.3×3.3 mm, field of view 211 mm, repetition time 2.16 s, echo time 25 ms, flip angle 75°, 36 slices, slice thickness 2.5 mm).

### Preprocessing

To preprocess the fMRI data, we use the statistical parametric mapping (SPM8, r613) software running on MATLAB (R2016b), together with the FIACH [24] package for R (3.2.2). First, we convert the data from DICOM to Nifti formats. We then delete the first four volumes of each session to avoid magnetic saturation effects. We subsequently re-align all images to the first remaining volume. To correct for possible artefacts, we apply the FIACH toolbox to the BOLD time series. We then normalise all data into standard MNI space with 2 mm isotropic voxels. All images are then spatially smoothed using a Gaussian filter of 8 mm fullwidth at half maximum. The BOLD signal is filtered between 0.04–0.07 Hz [25]. This frequency range is chosen to minimise the overlap between the BOLD signal and possible breathing and pulsation artefacts. We parcellate the brain into standard 90 automated anatomical labelling (AAL) regions (excluding the cerebellum) [26]. Finally, we apply principal component analysis (PCA) to the voxel time series within each region, from which we retain the first principal component to summarise the activity in each region [27].

Probabilistic tractography is used to preprocess DTI data using the iFOD2 algorithm within the MRtrix software [28]. Streamlines are filtered using SIFT [29], resulting in 10^7^ streamlines. We estimate a 90×90 structural connectivity matrix according to the number of streamlines connecting each pair of regions, normalised by their combined volumes. To reduce inter-subject variability, we then normalise each structural connectivity matrix relative to its maximum value. For each subject, we binarize the structural connectivity matrix according to 18 thresholds between 10% and 95% (in steps of 5%). Equipped with the structural measures *k*_*ij*_ and the summaries of regional activity in the seizure network, we estimate the latent states *x*_*i*_ and parameters of the DCM.

### Bayesian model inversion

To create a DCM of observable timeseries, we can use Eq (11) as a state space model with fast (analytic) fluctuations *ω*^(*i*)^ and map the latent states *x*_*i*_ to observable quantities with additive observation noise. Eq (11) is used to model all the neural time series presented in this paper, using standard (variational) routines in the Statistical Parametric Mapping (SPM) software. Specifically, we use generalised or variational Bayesian filtering (Dynamic Expectation Maximisation) [30]. This enables us to first estimate the parameters of the model for different patients. We then use the average of these parameters as a ground truth to generate data which we can then manipulate on a known circuit with plausible parameter values. In other words, with the DCM framework we aim to: a) infer the latent states; b) estimate the parameters; and c) estimate the hyperparameters, i.e. the precision components of fluctuations on the states and observation noise. After recovering the posterior densities, hyperparameters, and variational free energies on an individual level for all 30 subjects, we then obtain two group-level models–patients and healthy controls.

**Fig 3:**
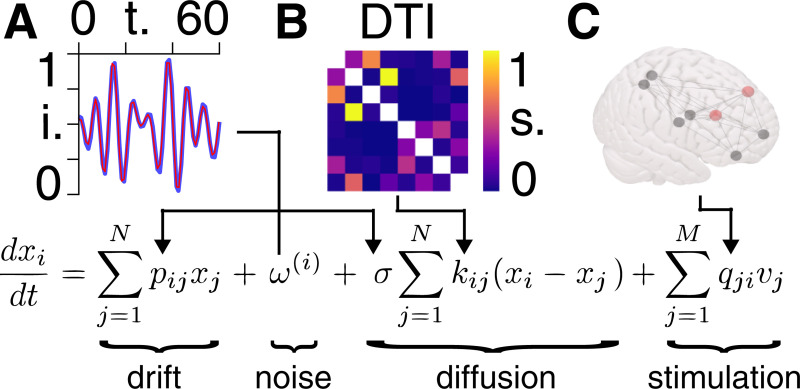
Equation of motion. **A)** BOLD signal intensity (i.), normalized between zero and unity, for an example time course (in seconds) in one patient (blue), together with the time series estimate (red) following Bayesian model inversion. This inversion provides posterior densities over the matrix elements p in the drift term, and the diffusion coefficient σ in the diffusion term, informed by a non-Markovian noise process ω. **B)** Example of an adjacency matrix from DTI data, from which we obtain matrix elements k in the diffusion term, following thresholding and binarization. **C)** The eight regions of the seizure network, in which the left and right frontal mid regions (red) give rise to unstable activity. Stimulation would then be applied to one or more of the remaining (black) regions in a way that mirrors the activity of the red nodes.

In Fig 3 we provide a visual aid to the governing equation of motion [11] in order to describe the meaning of each term. Note that, at this stage, we are not performing any gradient manipulation. The stochastic differential Eq (11) furnishes a dynamic causal model, where fast fluctuations are assumed to be small. This means that if the underlying dynamics can be described by Eq (11), then the model parameters *θ* = (*p*, *σ*) can be recovered from observations of BOLD signals *x* (Fig 3A).

As the parameter space associated with the eight-region seizure network is larger than can be accommodated by the time points available for each scan, we reduce the number of free parameters via functional connectivity priors [31,32]. This allows us to constrain the optimization such that we retain ~10× as many time points as free parameters. The adjacency matrix elements *k*_*ij*_ are not included as free parameters as they are known *a priori* from diffusion imaging (Fig 3B).) The system’s BOLD timecourses is measured in response to external stimulation *v*, which is supplied here in the form of EEG (Fig 3C).

### Bayesian model reduction

Bayesian model reduction is a type of model comparison, in which we calculate the free energy estimation to log model evidence *logp*(*d*|*m*) for the reduced model in which *σ* = 0 and the reduced posterior density over the *σ* parameter.

The free energy combines both accuracy and complexity when scoring models:

$$F=\underset{accuracy}{\underset{︸}{\langle \log\;p(d|\theta ,m)\rangle }}-\underset{complexity}{\underset{︸}{KL[q(\theta ),p(\theta |m)]}},$$ (21)

Here, *logp*(*d*|*θ*, *m*) is the log likelihood of the data *d* conditioned upon model states, parameters and hyperparameters *θ*, and model structure *m*. In this study we have two model structures: with diffusion and without diffusion; *q*(*θ*) is the approximate posterior density over *θ*; and *p*(*θ*|*m*) is the prior probability of *θ* given *m*, where the Kullback-Leibler divergence (*KL*) term allows for solutions *q*(*θ*) that are too complex to be penalised. In order to determine whether we are justified in including the diffusion term in the equation of motion [11], we first optimise the full model including a non-zero diffusion coefficient *σ* (i.e., including diffusion). We then use Bayesian model reduction [33,34] to estimate the evidence for the reduced model in which *σ* = 0 (i.e. excluding diffusion). The reduced model is specified by setting the prior variance over the diffusion coefficient *σ* to zero. The two models are compared by using the log Bayes Factor *F*(*σ* = 0)−*F*(*σ*≠0), which accounts for the relative evidence for non-diffusivity vs. diffusivity. Associated probabilities are then calculated by normalizing *F*, such that $p(\sigma =0)=\frac{F(\sigma =0)}{F(\sigma =0)+F(\sigma \neq 0)}$.

## Results

### Diffusivity is higher in the patient group

**Fig 4:**
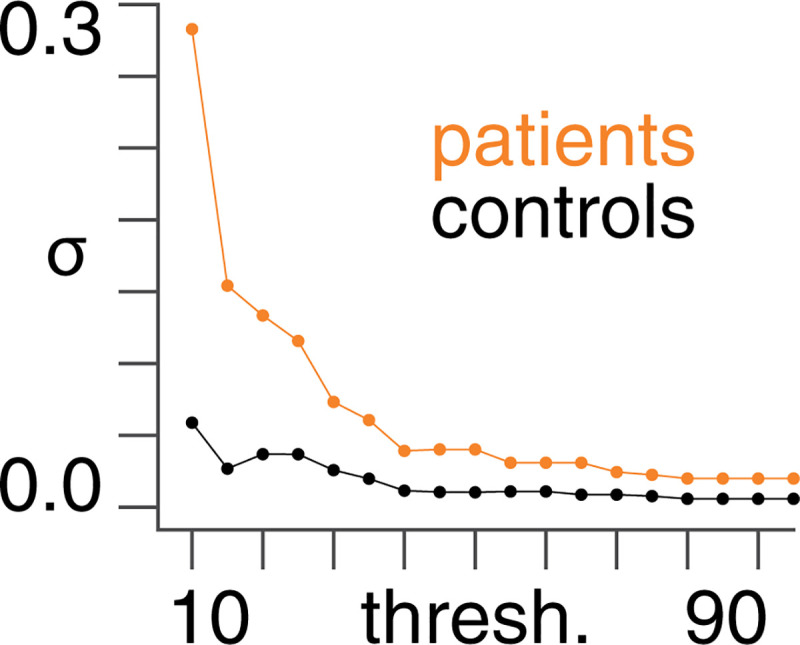
Diffusion coefficients. The diffusion coefficient (*σ*) (following Bayesian model averaging) as a function of structural (DTI) adjacency matrix threshold (%) for patients and controls. The 90% Bayesian credible intervals are sufficiently small to be contained within the data points.

We find that the patient group has higher diffusivity as compared with the control group across structural adjacency matrix thresholds used to define *k*_*ij*_ (see Fig 4).

The higher diffusivity in the patient group across all thresholds lends credence to the approach of suppressing seizure activity by decreasing diffusivity. We show the results in Fig 4 on an individual subject level in S1 Fig.

**Fig 5:**
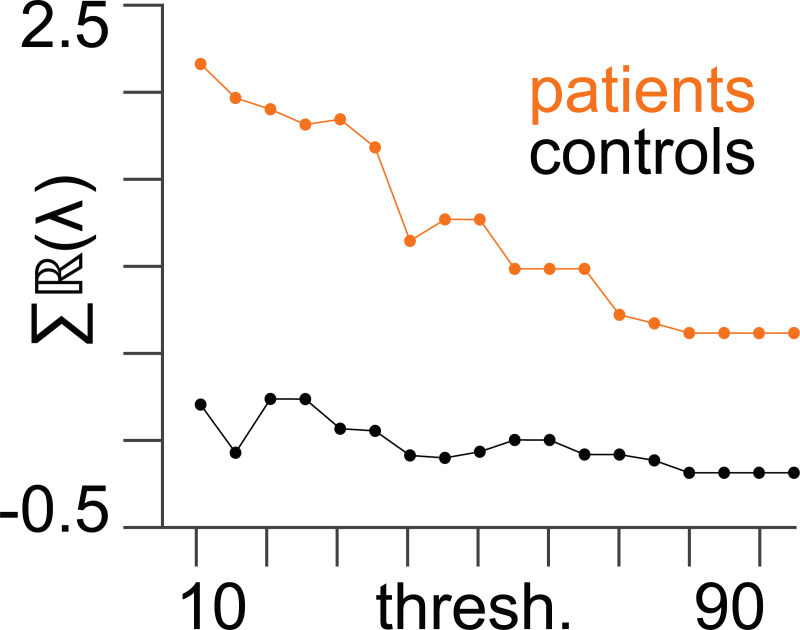
Stability. The sum of the Real components of the eigenvalues of the Jacobian (∑R(λ)) – measuring intrinsic stability of neural dynamics–as a function of structural (DTI) adjacency matrix threshold (%) for patients and controls following Bayesian model averaging. The 90% Bayesian credible intervals are sufficiently small to be contained within the data points.

Stability is lower in the patient group: We find that the patient group is less stable across structural adjacency matrix thresholds, as compared with the control group (see Fig 5). The lower stability in the patient group justifies the approach of aiming to increase stability. All subsequent results shown in this paper are calculated using a threshold of 50% to define structural connectivity (i.e., *k*_*ij*_). We show the results in Fig 5 on an individual subject level in S2 Fig.

### Accounting for diffusion improves models

**Fig 6:**
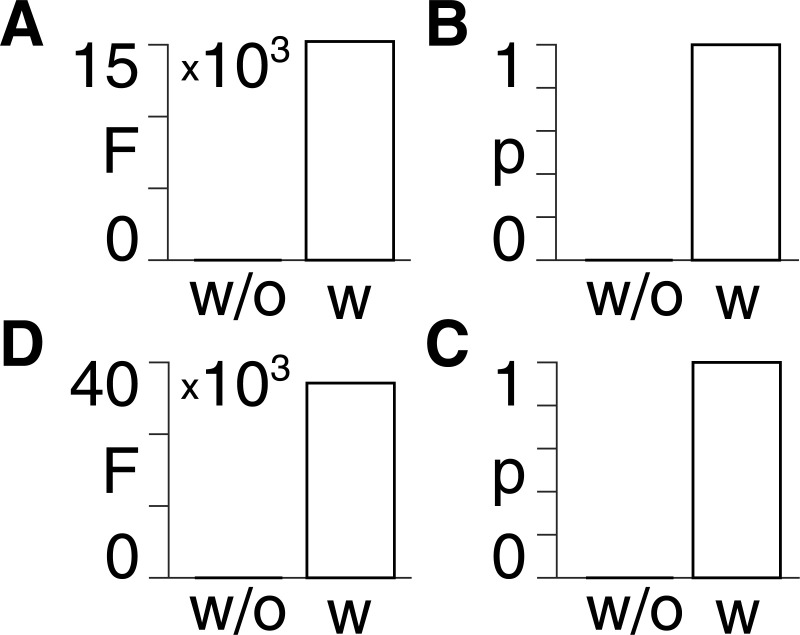
Bayesian model reduction. **A)** Control group. Approximate lower bound on log model evidence afforded by the free energy (F) following Bayesian model reduction for the reduced model without (w/o) diffusion and the full model with (w) diffusion. **B)** Probabilities derived from the log evidence in A). **C) & D)** Same layout as A) & B), but for the patient group.

We show, by using Bayesian model reduction (see Methods) in both patients and controls, that the variational free energies and associated probabilities are higher for the full models including diffusion, than in the reduced models excluding diffusion (Fig 6).

We show the results in Fig 6 on an individual subject level in S3 Fig.

This Bayesian model comparison provides evidence that the diffusion term in the equation of motion [11] gives a better description of the BOLD time series. Note that we do not increase the free energy automatically merely by adding another term. This would not be informative, as one can always improve model accuracy with more variables. Instead, the variational free energy describes a trade-off between accuracy and complexity. This means that the free energy can decrease if the complexity introduced by additional parameters is greater than the associated model accuracy increase. However, this is not the case here, in that we find that the increase in accuracy afforded by the diffusion term increases the variational free energy to a greater degree than the decrease caused by the heightened model complexity.

### Pre-ictal perturbation

**Fig 7:**
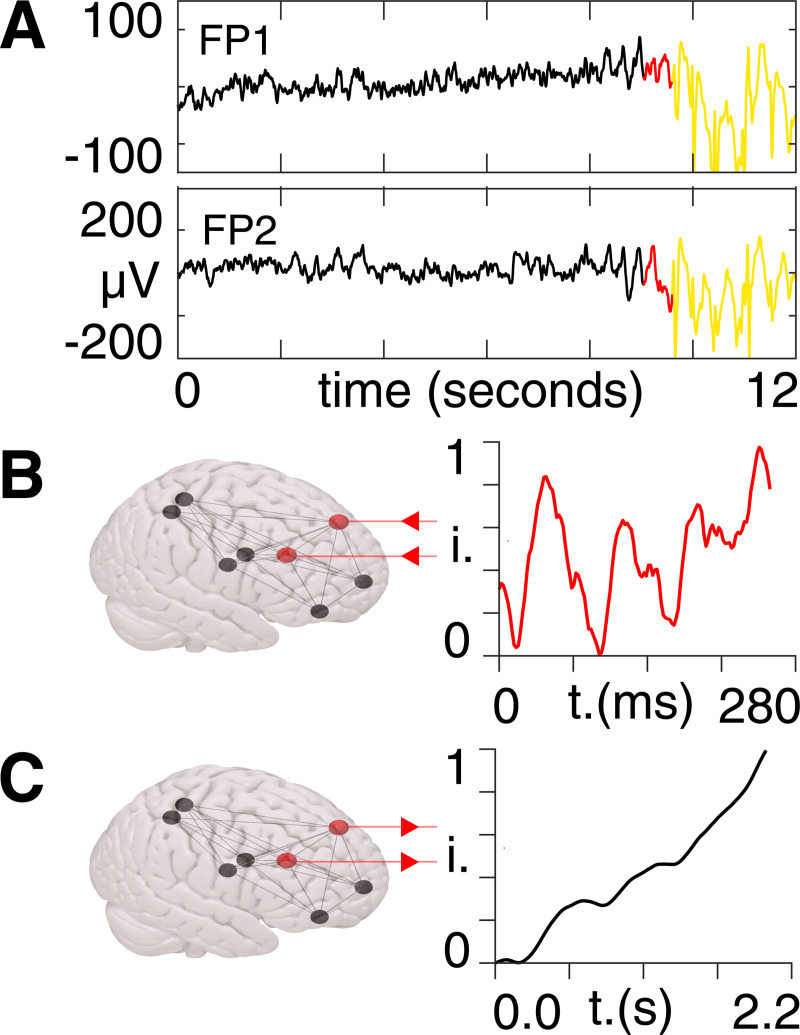
Pre-ictal perturbation. **A)** EEG activity from the frontal lobe (FP1 & FP2). Ictal activity is shown by the yellow section and pre-ictal activity is shown by the red section. **B)** The seizure network shown in MNI space (left) with the mid-frontal regions (left & right) indicated by the red nodes. The mean pre-ictal signal intensity (i) (right), normalized between zero and unity, corresponding to the red sections in A), is used as the external driving input and is supplied to the red nodes, as indicated by the inward-pointing red arrows. **C)** The mean BOLD signal intensity (i), normalized between zero and unity, of the mid-frontal region to the stimulus in B), as indicated by the outward-pointing red arrows.

Following Bayesian model inversion and averaging in the patient group, we use the resulting parameters to create an *in silico* seizure network, in which we perturb the mid-frontal sources with pre-ictal activity taken from EEG measurements (Fig 7A and 7B).

We use the mean activity from EEG channels located close to the frontal region (FP1 & FP2), as these pick up a mixture of frontal source activities. Note that previous studies have indicated the vital role of the frontal lobe in JME [35,36]. Prior to GSW discharge events, we observe a change in the EEG channels exclusively in the frontal lobe region, with typical background activity seen in other channels. Following this stimulation, we see that the activity profiles from the mid-frontal regions climb in an uncontrolled manner, due to the associated eigenvalues with positive Real components (Fig 7C). Note, however, that which regions are stable or not varies across patients and that stability, or lack thereof, does not imply anything about focal pathology–given that we are dealing with generalized epilepsy.

### Seizure suppression

**Fig 8:**
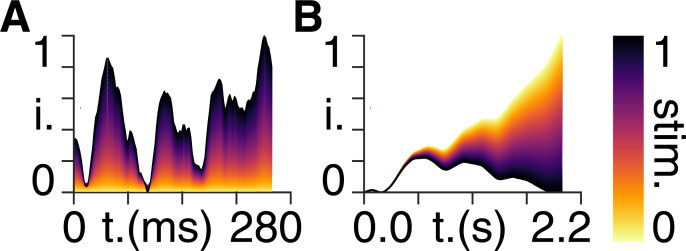
Simulating seizure suppression. **A)** External stimulation signal intensity (i), normalized between zero and unity, applied to all nodes except for the mid frontal region for 1000 forward models ranging from zero stimulation (yellow) to a stimulation profile that perfectly mirrors the pre-ictal activity in Fig 7B (black). **B)** Response signal intensity (i), normalized between zero and unity, of the mid frontal region for the same 1000 forward models in A) with matching colours, i.e. the yellow response corresponds to zero stimulation and the black response corresponds to a stimulation profile that perfectly mirrors the pre-ictal activity in Fig 7B.

Using the same setup as in Fig 7, we again run the forward model. However, this time–in addition to the pre-ictal driving stimulus–we supply additional external stimulation to all nodes *except* the mid-frontal region, in a way that mirrors the pre-ictal activity in Fig 7B. Note that, as stability is determined purely by the Jacobian of the system, this perturbation does not alter the stability in the regions. Rather, this pre-emptive intervention induces a temporary ‘pseudo stability’, in which the activity of an affected region can be driven back to baseline. We show that, in agreement with our theoretical predictions, reducing activity gradients in this way results in the response of the unstable mid-frontal region being suppressed (Fig 8).

We show an animated version of Fig 8 in S1 Movie.

We note from Fig 8 that the methodology we are proposing works best when the input exactly matches the signal, i.e. with minimized gradients. This can be seen by the gradual shift in the response as the stimulation becomes increasingly similar to the signal–from an initial divergence to baseline recovery. Note also that, although these simulations are based on fMRI data collected in patients with epilepsy, these patients did not experience seizures while in the scanner: the pre-ictal activity collected with EEG was recorded separately. The output of the model in Figs 7C and 8B should therefore not be viewed as mimicking a seizure-like BOLD response. Instead, what we show here is that, in response to a perturbation in the form of real pre-ictal activity (Figs 7B and 8A), an initially unstable system with a diverging response can be suppressed–a result that we suggest could be clinically advantageous in the treatment of epilepsy.

### Minimum effective stimulation

In the previous section we stimulated all nodes except for the mid-frontal region to demonstrate proof of principle. However, for treatment purposes, it is clearly better to stimulate as few regions as possible, in order to remain minimally invasive. We therefore proceed by using each individual node in turn as the stimulation target. This allows us to determine the order of the regions, ranked in terms of the lowest stimulation strength required to suppress the mid-frontal response (Table 1).

**Table 1:** Ranking order in terms of the stimulation strength required to suppress the BOLD response in the mid-frontal region to the same extent as in Fig 8B, by stimulating a single region in the seizure network. Stimulation strengths are presented relative to the lowest value (thalamus left), which is assigned a value of unity. N/A values are assigned if it is not possible to suppress the BOLD response in the mid-frontal region by targeting the corresponding single region.

Rank	Region	Stimulation
**1**	Thalamus (left)	1
**2**	Frontal Mid Orbital (right)	20
**3**	Precuneus (left)	76
**4**	Precuneus (right)	82
**5**	Frontal Mid Orbital (left)	N/A
**6**	Thalamus (right)	N/A

Note that the mid-frontal region is not included in this ranking as it is being supplied with the driving input in the form of pre-ictal activity and our technique requires mirroring this activity in neighbouring nodes in the network, in order to decrease gradients and thus diffusivity. Using this *in silico* stimulation protocol, we find that the thalamus (left) requires the lowest stimulation strength (by a considerable margin) in order to suppress activity in the unstable mid-frontal region, relative to the other six regions in the seizure network.

## Discussion

We began by showing that the linear DCM neuronal state equation takes the form of the Fokker-Planck equation when generalised to account for the propagation of neuronal activity over structured connections. We then demonstrated that the Fokker-Planck equation provides an accurate description of the evolution of neural signals. This empirical evidence, together with the fact that the Fokker-Planck equation is a statement of the conservation of probability [37], allows us to infer that there should exist a conservation of neuronal activity in the brain. This inference can be plausibly motivated in terms of conservative aspects of neuronal message passing, such as the balance between excitation and inhibition [38,39]. This balance lies at the base of functional modes, which are found (particularly in the cortices) to repeat across scales in the brain [40]. The canonical computational units at the most elementary scale take on various ratios of excitatory and inhibitory neurons. However, the current consensus is that the basic unit of the cortical system is the pyramidal interneuron gamma network (PING) [41], which is made up of a pyramidal excitatory neuron (PN) and a fast spiking inhibitory parvalbumin interneuron (IN). One can interpret the diffusion term in Eq (11) as an intrinsic mode of interaction between such neuronal units across spatial scales, where the responsible mechanism could be due to: a) neurotransmission at the microscopic scale of individual neurons, b) electrical impulses at the mesoscopic scale of microcircuits, or c) long-range white matter connections between brain regions at a macroscopic scale.

The intervention technique we are proposing is a departure from the status quo, which usually involves the opposite approach–namely, increasing inhibition [42–44]. The signs of *p*_*ij*_ in Eq (11) are allowed, in the subsequent Bayesian model inversion, to be either positive or negative. Therefore, depending on the values of the diffusion coefficient *σ* and the structural adjacency matrix *k*_*ij*_, a node could be either inhibitory or excitatory–i.e. with a negative or positive eigenvalue, respectively. However, we do not influence the sign of the eigenvalues with our proposed technique, we only regulate the difference between activity levels. In other words, if a system is e.g. inhibitory to begin with then we do not change this–we only change the extent to which this inhibition can spread throughout the system via gradient manipulation. Our method was shown to work in the context of forward generative models, parameters of which were optimised from data collected in epilepsy patients. Specifically, we applied stimulation in such a way as to decrease activity gradients between unstable regions and their neighbouring nodes, thereby decreasing diffusivity. It should be noted that the diffusion-driven method shown here could be applied to *any* disorder that would benefit from either a decrease or increase in diffusivity–in other words, this method is not specific to epilepsy. For instance, it may be beneficial to use the same premise in the treatment of e.g. disorders associated with cortical spreading depression (CSD) [45].

There are a number of assumptions upon which the theoretical premise of this paper are based that should be kept in mind for future practical implementation of the proposed techniques. The most basic of these underlying assumptions is that the recorded voltage is the primary pathological signal that propagates via diffusion. This assumption is widely accepted, but a theoretical treatment such as the one present here ignores potential bystander effects (e.g. ions, glia etc.) which may in fact contribute as active factors. The next most basic assumption is that the recorded activity can be adequately described by an LTI as shown in Eq (11). The advantages of using this kind of linear approximation lies within the associated computational expediency. However, using the same methods presented it would be equally possible to use a Taylor series expansion to any arbitrary number of terms in order to capture higher-order interactions [46]. It should here be noted that, regardless of how many terms are included to capture non-linearities, stability (or lack thereof) will only be determined by the Jacobian of the system and not by any external perturbations–a central premise of the work presented here.

A potentially more problematic point with regard to the underlying model is that its parameters are furnished following Bayesian model inversion on long timescale datasets in non-seizure states. However, these same models may then not be applicable in seizure states, in which completely different pathways are activated. This is one of the reasons why we suggest that our proposed methodology be applied pre-emptively, preferably long before the pre-ictal activity sets in, as otherwise the model upon which the intervention is based may be invalidated by changes in physiology brought on by seizure activity.

A further assumption upon which our study is based is that the DCM neuronal state equation can be generalised via a minimally axiomatic premise to account for structural connectivity. This is an assumption that can be falsified empirically by testing whether the additional diffusion term increases or decreases model evidence–as shown here using Bayesian model reduction (see Methods). This same type of analysis should be performed on any dataset prior to further analysis in order to make sure that the generalisation is beneficial for the application in question. Following this initial check, it is then assumed that the underlying dataset can be modelled using the Fokker-Planck formalism.

We also proceed under the premise that the gradients are the only aspect of the system that can be altered, i.e. we cannot change the structural adjacency matrix or the diffusion coefficient. The reason for this premise is that we wish to facilitate minimally invasive techniques that do not involve severing structural connections, e.g. via surgical intervention. There may, however, be situations in which changes to the adjacency matrix need to be taken into account–for instance if contemplating the type of electrical stimulation we are proposing in conjunction with surgery.

Overall, the approach proposed here provides a novel framework to address the three fundamental questions of *when*, *where*, and *how* to stimulate the brain in order to suppress pathological activity in the context of epilepsy. In particular, we investigated the impact of stimulation strength in relation to the number of stimulation sites and proposed specific timings and profiles. As our technique relies upon supplying otherwise healthy regions of the brain with stimulation that mirrors pathological activity, it is clearly clinically advantageous to target as few regions as possible. It is for this reason that we focused on targeting a single region and found that the thalamus required the lowest stimulation strength, in line with the broad literature showing the thalamus to be a key region in seizure generation [47–50]. However, it is in principle possible to target multiple nodes. The trade-off between the number of target nodes and stimulation strengths required will need to be determined on a patient-by-patient basis. The goal is to allow for as many patients as possible to benefit from a non-invasive technique that could limit the need for either surgical intervention or pharmacological treatments with undesirable side effects.

Stimulation techniques that can generate arbitrary waveforms are currently available using both invasive and non-invasive methods. The fast computational processing times associated with our strategy render it compatible with closed-loop approaches, which are increasingly seen as providing the greatest clinical efficacy in delivering personalised treatment [51]. There are similarities between our approach and coordinated reset strategies, given that our results support targeting several stimulation sites in a spatiotemporally coordinated manner [52,53]. However, a critical difference to existing methods is that we demonstrate that abnormal activity can be mitigated by *increasing* activity in a strategic manner, rather than by following a phase-resetting mechanism. Specifically, we demonstrate that unstable activity can be suppressed by modulating the functionally connected neighbours of affected brain regions. The stimulation profile and timing are chosen in such a way as to mirror the activity of the pathological region (e.g. the epileptogenic zone) prior to seizure onset. We demonstrated application using pre-ictal activity, but in practice it may be more advantageous to stimulate pre-emptively on a continual basis–ideally preventing the seizure from ever occurring.

## Supporting information

S1 Fig Diffusion coefficients per subject. All healthy controls (H) and patients (P). For all figures: the diffusion coefficient (σ) as a function of structural (DTI) adjacency matrix threshold (%) for patients and controls.
(EPS)

S2 FigStability per subject.All healthy controls (H) and patients (P). For all figures: The sum of the Real components of the eigenvalues of the Jacobian (∑R(λ)) – measuring intrinsic stability of neural dynamics–as a function of structural (DTI) adjacency matrix threshold (%) for patients and controls.(EPS)

S3 Fig Bayesian model reduction per subject. All healthy controls (H) and patients (P). For all figures: **Left:** Approximate lower bound on log model evidence afforded by the free energy (F) following Bayesian model reduction for the reduced model without (w/o) diffusion and the full model with (w) diffusion **Right:** Probabilities derived from the log evidence.
(EPS)

S1 Table Demographics of the subjects in this study. (XLSX)

S1 Movie Animated version of Fig 8, with each incremental step in external stimulation shown for the 1000 forward models. (MOV)
